# Anterior Cingulate Cortico-Hippocampal Dysconnectivity in Unaffected Relatives of Schizophrenia Patients: A Stochastic Dynamic Causal Modeling Study

**DOI:** 10.3389/fnhum.2016.00383

**Published:** 2016-07-27

**Authors:** Yi-Bin Xi, Chen Li, Long-Biao Cui, Jian Liu, Fan Guo, Liang Li, Ting-Ting Liu, Kang Liu, Gang Chen, Min Xi, Hua-Ning Wang, Hong Yin

**Affiliations:** ^1^Department of Radiology, Xijing Hospital, Fourth Military Medical UniversityXi’an, Shaanxi, China; ^2^Network Center, Fourth Military Medical UniversityXi’an, Shaanxi, China; ^3^School of Biomedical Engineering, Fourth Military Medical UniversityXi’an, Shaanxi, China; ^4^Department of Psychiatry, Xijing Hospital, Fourth Military Medical UniversityXi’an, Shaanxi, China

**Keywords:** schizophrenia, first-degree relatives, functional magnetic resonance imaging, effective connectivity, stochastic dynamic causal modeling

## Abstract

Familial risk plays a significant role in the etiology of schizophrenia (SZ). Many studies using neuroimaging have demonstrated structural and functional alterations in relatives of SZ patients, with significant results found in diverse brain regions involving the anterior cingulate cortex (ACC), caudate, dorsolateral prefrontal cortex (DLPFC), and hippocampus. This study investigated whether unaffected relatives of first episode SZ differ from healthy controls (HCs) in effective connectivity measures among these regions. Forty-six unaffected first-degree relatives of first episode SZ patients—according to the DSM-IV—were studied. Fifty HCs were included for comparison. All subjects underwent resting state functional magnetic resonance imaging (fMRI). We used stochastic dynamic causal modeling (sDCM) to estimate the directed connections between the left ACC, right ACC, left caudate, right caudate, left DLPFC, left hippocampus, and right hippocampus. We used Bayesian parameter averaging (BPA) to characterize the differences. The BPA results showed hyperconnectivity from the left ACC to right hippocampus and hypoconnectivity from the right ACC to right hippocampus in SZ relatives compared to HCs. The pattern of anterior cingulate cortico-hippocampal connectivity in SZ relatives may be a familial feature of SZ risk, appearing to reflect familial susceptibility for SZ.

## Introduction

It is well established that familial risk plays a significant role in the etiology of schizophrenia (SZ) through family, adoption, twin, and sibling studies. SZ as a hereditary component affects 0.3% to 0.7% of the general population globally according to American Psychiatric Association (APA, 2013), whereas first-degree relatives have a higher risk of developing SZ, with an actual prevalence of approximate 10% (Lim and Sim, 1992). In genetic epidemiology studies, a 31% to 58% concordance rate of SZ exists in monozygotic twins (Tsuang, 2000). It has been demonstrated that genetic liability to SZ was 81% (95% confidence interval (CI): 73%, 90%) based on results from 12 twin studies of SZ (Sullivan et al., 2003). The individual’s heritability in liability just partly mediates family history of SZ (Agerbo et al., 2015). Furthermore, brain structural deficits in twins discordant for SZ were more pronounced in monozygotic than in dizygotic twins (Baare et al., 2001; Hulshoff Pol et al., 2004, 2006), suggesting association of cerebral abnormalities with genetic factors for SZ. In our previous studies, we have detected altered brain structure and function in first episode drug-naïve SZ patients (Chang et al., 2015; Cui et al., 2015, 2016; Huang et al., 2015). Thus the question is whether their first-degree relatives present specific alterations of the brain.

During the past 5 years, many structural magnetic resonance imaging (MRI) studies have revealed that gray matter volume, cortical morphological features, and white matter integrity in individuals at high risk of SZ differ from controls, but usually to a lesser extent than in SZ patients, indicating that structural aberrancies may form markers of susceptibility and transition to this disease (Bois et al., 2015b), despite not absolutely consistent findings. For the cerebral morphology, an interrupted cingulate sulcus pattern and paracingulate morphology are associated with increased genetic risk of SZ (Meredith et al., 2012). In the Edinburgh High Risk Study by Lawrie et al. cortical thinning pronounced in the left middle temporal gyrus (Sprooten et al., 2013), as well as longitudinal reductions for volume of the whole brain and bilateral prefrontal and temporal lobes (McIntosh et al., 2011) and cortical surface area prominently in the frontal, cingulate, and occipital lobes (Bois et al., 2015a) were detected in individuals at familial high risk of SZ compared with controls. Also, young relatives of SZ patients showed reduced bilateral hippocampal volume (Thermenos et al., 2013). As reported in a meta-analysis by Cooper et al. (2014), the gray matter volume increased in the left middle frontal gyrus, and decreased in the left thalamus/putamen, insula, and right superior frontal gyrus in high-risk individuals.

With the exception of diverse structural abnormalities, overall, a series of studies have demonstrated functional alterations in relatives of SZ patients at resting state (McIntosh et al., 2006; Hao et al., 2009; Jang et al., 2011; Liao et al., 2012; Su et al., 2013; Zhou et al., 2015) or task state (Whitfield-Gabrieli et al., 2009; Woodward et al., 2009; Rasetti et al., 2011; Stolz et al., 2012), with significant results found in several specific brain regions involving the dorsolateral prefrontal cortex (DLPFC), anterior cingulate cortex (ACC), caudate, and hippocampus.

Notably, the left DLPFC is a featured brain area in SZ relatives. It has been found that familial liability to SZ was associated with decreased gray matter volume of the left DLPFC (McIntosh et al., 2006). Furthermore, healthy siblings of SZ patients showed reduced white matter fractional anisotropy (FA) in the left DLPFC, without significant difference between SZ patients and their siblings (Hao et al., 2009). DLPFC dysfunction has been implicated in the familial susceptibility for SZ (Li and Funahashi, 2015). Aberrant regional function of the left DLPFC was detected by a resting state MRI study on the first-degree relatives of SZ patients (Liao et al., 2012). When identifying familial vulnerability markers by examining default mode network (DMN) connectivity, posterior cingulate cortex (PCC) seed region connectivity analysis showed reduced functional connectivity in the bilateral DLPFC of relatives (Jang et al., 2011). Unaffected relatives also had impaired connectivity from the left DLPFC to its coordinated regions, distributed in the bilateral caudate, left middle frontal gyrus, and right cerebellum (Su et al., 2013). However, few studies examined connectivity between some of these brain regions in unaffected relatives of SZ patients (Meda et al., 2012; Su et al., 2013), to date, leaving the open question of brain connectivity among these areas in familial high risk individuals.

Although previous studies have identified brain structural and functional abnormalities in frontal and temporal regions, it is still unclear how these regions interacts with each other differently in relatives of SZ patients compared with healthy controls (HCs). In the current study, we used stochastic dynamic causal modeling (sDCM) to investigate directed brain connectivity within a brain network encompasses ACC, caudate, DLPFC and hippocampus. DCM is a technique to investigate brain effective connectivity which refers to the causal influence of one brain region exerts over another or itself (Friston et al., 2003). Compared with functional connectivity analysis which simply measures the correlations between the blood-oxygen-level-dependent (BOLD) signals of different brain regions, effective connectivity analysis is able to further provide us information on how the signals are propagated within a brain network. Understanding the information flow within a brain network is crucial for understanding the neural mechanism of familial susceptibility for SZ. DCM was first invented to model the interactions between brain regions during task performance (Friston et al., 2003). Dauvermann et al. (2013) found decreased thalamo-cortical connectivity in first- or second-degree relatives of SZ patients using nonlinear deterministic DCM during verbal fluency processing. Recently, traditional deterministic DCM has been extended to stochastic DCM (Daunizeau et al., 2009; Li et al., 2011, 2014) which is also able to model brain effective connectivity at rest (Li et al., 2012). Here we used sDCM to identify changes in brain effective connectivity in unaffected first-degree relatives of SZ patients using resting-state fMRI (rsfMRI) data. On the basis of existing evidence that familial risk for SZ appears along with aberrant brain structural and functional alterations involving DLPFC, ACC, caudate, and hippocampus, we hypothesized that effective connectivity among them would also be disrupted in relatives, and provide more accurate parameter estimates (Li et al., 2011) compared with conventional deterministic DCM.

## Materials and Methods

### Subjects

We assessed 53 HCs and 48 unaffected first-degree relatives of patients with first episode SZ (age- and gender-matched to HCs). The Diagnostic and Statistic Manual of Mental Disorders, 4th edition (DSM-IV), revised criteria (Mittal and Walker, 2011) consensus diagnoses were established by two trained senior clinical psychiatrists with all clinical data and Structured Clinical Interviews for DSM Diagnoses interviews: inter-rater reliability was higher than 90% among raters. Relatives of probands were free of Axis 1 psychopathology and not taking psychoactive medications. Participants were recruited via word of mouth and advertisements at the Fourth Military Medical University; all provided written informed consent approved by the institutional review board of Xijing Hospital.

### Data Acquisition and Preprocessing

**Figure 1:**
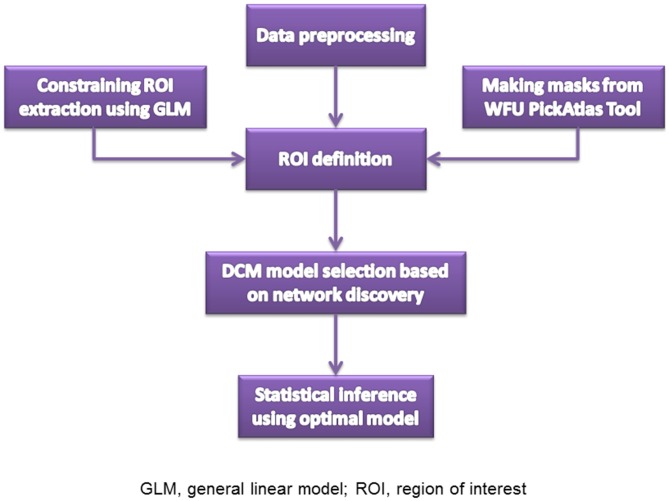
**Steps for data analysis**.

The resting state fMRI images were collected on the 3.0-T Siemens Magnetom Trio Tim scanner. High-resolution T1-weighted 3D anatomical data were acquired using the 3D magnetization-prepared rapid gradient echo (3D MPRAGE) sequence (repetition time (TR): 2530 ms; echo time (TE): 3.5 ms; flip angle: 7°; field of view (FOV): 256 × 256 mm^2^; matrix: 256 × 256; slice thickness: 1 mm; section gap: 0 mm; number of slices: 192). The image resolution was 1 mm × 1 mm × 1 mm. The echo planar imaging (EPI) sequence (TR: 2000 ms; TE: 30 ms; flip angle: 90°; FOV: 220 × 220 mm^2^; matrix: 64 × 64; slice thickness: 4 mm; section gap: 0.6 mm) effectively covered the entire brain. Head motion was restricted with a custom-built head-coil foam cushion. During scanning, participants were asked to remain alert with eyes closed and head still. These instructions aided reducing head motion and prevented subjects from falling asleep. All participants were judged as awake and alert at the start and conclusion of the fMRI session. Figure 1 is the flowchart for each step. Images were reconstructed offline, and realigned with statistical parametric mapping (SPM8^1^). The translation/rotation corrections of each participant were examined to exclude excessive head motion (>2.5 mm translation and/or >2.5° rotation), resulting in that eventual 46 first-degree relatives of SZ patients and 50 HCs were included. A mean functional image volume was constructed for each session from the realigned image volumes to determine parameters for spatial normalization into Montreal Neurological Institute standardized space^2^. Normalization parameters determined for the mean functional volume were applied to the corresponding functional image volumes of each participant, which were smoothed with an 8 mm full width half maximum (FWHM) Gaussian kernel.

### General Linear Model

In the first-level (within subject) analyses, participant-specific responses were modeled using a general linear model (GLM). The six motion parameters were included to model the movement correlated effects. One constant regressor was used to model the baseline, and cosine basis functions were included in the GLM. The resulting contrast images were then used to constrain the region of interest (ROI) extraction step in the sDCM.

### Stochastic Dynamic Causal Modeling

#### Regions of Interest

**Figure 2:**
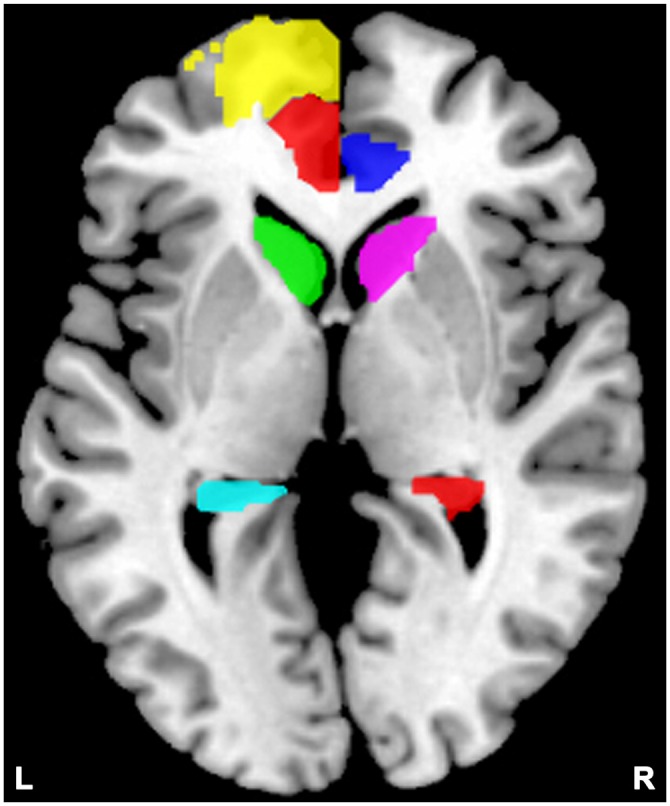
**Locations of the masks.** Yellow indicates the left dorsolateral prefrontal cortex (DLPFC); semitransparent red indicates the left anterior cingulate cortex (ACC), and blue indicates the right ACC; green indicates the left caudate, and violet indicates the right caudate; cyan indicates the left hippocampus, and red indicates the right hippocampus.

For each subject, we studied the effective connectivity among seven ROIs including the left DLPFC (consists of Frontal_Sup_L and Frontal_Sup_Medial_L), and the bilateral ACC (Cingulum_Ant_L and Cingulum_Ant_R), caudate nuclei (Caudate_L and Caudate_R), and hippocampi (Hippocampus_L and Hippocampus_R). The left rather than the right DLPFC showed alterations in most studies of SZ relatives during rest condition (McIntosh et al., 2006; Hao et al., 2009; Liao et al., 2012; Su et al., 2013) thereby being chosen as the ROI. For each region, a ROI mask of that region was created by the WFU PickAtlas Tool (Version 3.0.4^3^) and the automated anatomical labeling (AAL) atlas template (Figure 2; Tzourio-Mazoyer et al., 2002; Maldjian et al., 2003, 2004). Subject-specific time series were then extracted based on the ROI mask and the contrast image generated by first-level (within subject) analyses. We then extracted time series from the voxels within the ROI that also showed activation in the contrast image. The first principle component of these time series was finally used to summarize the BOLD response to the ROI.

#### Model Specification and Parameter Estimation

**Figure 3:**
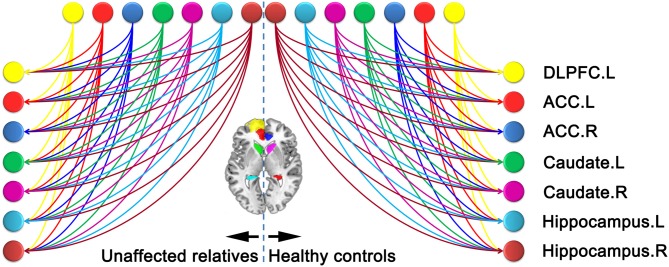
**Fully connected model constructed.** The lines with arrowheads between distinct region of interests (ROIs) refer to the connections in the left panel for relatives of schizophrenia (SZ) patients and right panel for healthy controls (HCs). The color of each node is in line with that of Figure 2. ACC, anterior cingulate cortex; DLPFC, dorsolateral prefrontal cortex.

In the current study, we aimed to search over all possible models generated from the connections among the seven ROIs. In this case, we did not limit our analysis to simply compare a few competing hypothesis (models). In contrast, we used a data-driven approach to search over all possible models. Specifically, a fully connected model (full model) with bidirectional connections between any pair of regions was constructed for each subject (Figure 3). Parameter estimates and model evidence of the full model was obtained using generalized filtering which is a recently developed scheme for sDCM model inversion and parameter estimation (Friston et al., 2010). After the full model was inverted, we employed a network discovery procedure (Friston et al., 2011) to search for the best reduced model which has the highest model evidence. A reduced model has the same group of ROIs as the full model, but only a subgroup of the connections in the full model (i.e., some of the connections are absent in the reduced model). The network discovery scheme provides approximation of the model evidences of all the possible reduced models without inverting every reduced model. The reduced models and the full model are then scored according to their model evidence. Model which has the highest model evidence was chosen as the winning model. Parameter estimates of the winning model were also obtained using the network discovery scheme and used for group analysis and making inferences on effective connectivity between brain regions.

#### Group Analysis

On the basis of sDCM analysis, the strength of connection described the coupling strength according to the rate at which neuronal responses were triggered in the target area (connection strengths are effectively rate constants in 1/s, Hz; Friston et al., 2003). To see whether these differences could be estimated and detected reliably, we characterized the differences using Bayesian parameter averaging (BPA; Friston et al., 2014; Razi et al., 2015). We used BPA for each group separately after network discovery procedure. We can then go on to discuss the results based on largest two or three connection differences, thereby being as a guiding principle to set the threshold (strength of connections measured in Hz).

## Results

### Demographical Characteristics

No significant differences were present between SZ patients’ first-degree relatives and HCs on any demographic variables (Table 1).

**Table 1:** **Demographical data of the participants**.

Variables	First-degree relatives of SZ patients	HCs	Statistics	*P* value
Age (years)	28 ± 5	27 ± 4	*t* = −0.35	0.73
Gender (M/F)	22/24	31/19	*χ*^2^ = 1.95	0.22
Ethnicity	Han (Chinese)	Han (Chinese)	—	—
Handedness (R/L)	46/0	50/0	—	—
Education (years)	15 ± 1	15 ± 2	*t* = 0.23	0.82
Smoking status (S/N)	11/35	18/32	*χ*^2^ = 1.66	0.27

*M, male; F, female; R, right; L, left; S, smoker; N, nonsmoker*.

### Network Discovery-Based Model Selection Results

**Figure 4:**
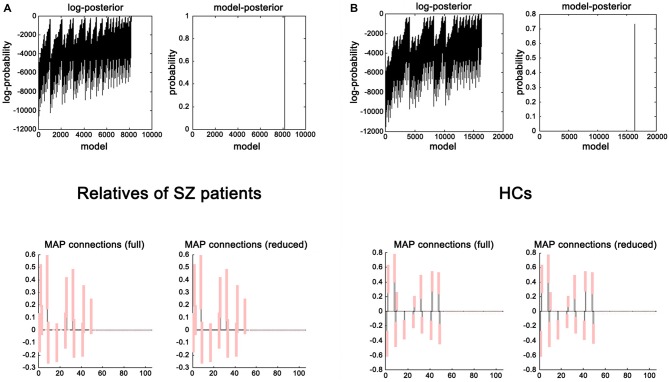
**Results of the *post hoc* optimization.** The corresponding conditional parameter estimates were shown over the 49 (extrinsic and intrinsic) connections in relatives of SZ patients **(A)** and HCs **(B)**. This figure suggested that the fully connected model was the best explanation for the data.

The evidence of all reduced models was compared by the network discovery procedure for each group (Figure 4). The left panel is for first-degree relatives of SZ patients and right panel refers to HCs. The procedure selected the fully connected model as the best model with a posterior probability of almost 1. The fully connected model had 49 parameters describing the extrinsic connections between nodes and the intrinsic (self-connections) within nodes. In Figure 4, the profiles of model evidences are shown with the posterior probability for each model. In both groups, the full model had a log-probability of almost 0 and probability of 1. Therefore, they shared the identical winning model.

### Effectivity Connectivity

**Figure 5:**
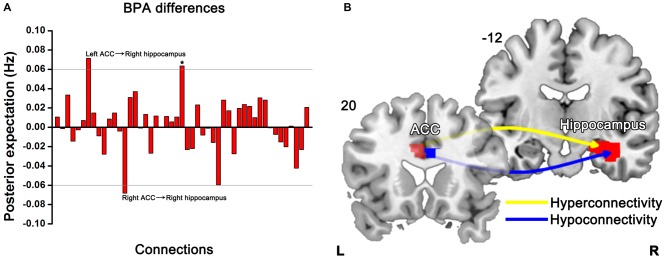
**Significant effective connectivity (between the group level) among ROIs in the first-degree relatives of SZ patients and HCs.** Bayesian parameter averaging (BPA) of the differences for stochastic dynamic causal modeling (sDCM) shows only those edges on the graph that survive the threshold of 0.06 Hz, i.e., the increased (left ACC-right hippocampus) and decreased (right ACC-right hippocampus) connections in relatives compared to HCs **(A)**. Schematic illustration showing connectivity patterns in first-degree relatives of SZ patients **(B)**. *Indicates self-connection of the left caudate. The slice location (coordinate) is marked in the upper-left.

BPA results of the effective connectivity can be seen in Figure 5. When using BPA, in the context of uncovering the group differences, as a guiding principle it would be best to choose top two or three connections and then we set the threshold to 0.06 Hz. SZ patients’ relatives exerted increased connection from the left ACC to right hippocampus, but decreased connection from the right ACC to right hippocampus as compared to HCs.

## Discussion

Our study presents sDCM-based effective connectivity outcomes contrasted between first-degree relatives of first episode SZ patients and HCs. As compared with HCs, first-degree relatives who did not show any psychiatric symptoms revealed abnormal connectivity primarily localized to the connections from the bilateral ACC to right hippocampus.

Cognitive deficits are a core characteristic of SZ (Elvevag and Goldberg, 2000), which has been previously observed in biological relatives of SZ patients (Snitz et al., 2006; Bove, 2008; Keshavan et al., 2010; Liao et al., 2012). As well, impaired neural circuitry within the emotion processing has been reported in unaffected siblings of SZ patients (van Buuren et al., 2011; Hanssen et al., 2015). The neural basis of impaired cognition, including emotion processing, in SZ patients and their relatives remains uncertain, thus leaving an open question of whether presence of cognitive and emotional deficits in unaffected first-degree relatives at high risk for developing SZ suggests genetic basis of SZ symptoms. Determining the neural correlates of familial risk for SZ is essential to elucidate the neurobiology for SZ that may aid in the development of novel targeted treatment.

For one thing, relatives of SZ patients exhibited bilateral anterior cingulate cortical dysconnectivity in our present study. Previous studies have demonstrated the important role of the dorsal ACC in cognitive control (Carter and van Veen, 2007) consistently. For these regions, these are association of aberrant activation patterns with deficient behavioral performance in SZ (Minzenberg et al., 2009). Most recently, we found altered effective connectivity related to ACC in SZ patients using spectral DCM, indicating anterior cingulate cortico-prefrontal-hippocampal hyperconnectivity (Cui et al., 2015). The effective connectivity and white matter connectivity analysis provides some evidence that weaker connectivity involved in ACC may be the neural basis of specific cognitive impairments in SZ (Wagner et al., 2015). Furthermore, using a regional homogeneity approach, Liao et al. (2012) reported decreased local neural activity in ACC in first-degree relatives of SZ patients along cognitive deficits. When taken with these previous results, our findings in unaffected relatives point to the possibility of altered functional interplay between ACC and hippocampus as the unit responsible for cognition and initial sign for developing SZ.

For another, we detect abnormal connection from ACC to the right hippocampus in relatives of SZ patients. Reduced bilateral hippocampal volume has been observed in young relatives of SZ patients (Keshavan et al., 2002; Thermenos et al., 2013). It has been found smaller hippocampi in relatives of SZ patients (Seidman et al., 2002, 2014; Francis et al., 2013). Furthermore, patients with SZ and their healthy siblings shared disrupted white matter integrity in the hippocampus that may be related to higher risk of healthy siblings to develop SZ (Hao et al., 2009). The hippocampus is part of the hippocampal formation that is comprised of subfields namely the dentate gyrus, subiculum, and pre-subiculum. By means of Van Leemput et al. (2009) method enabling quantification of elusive subfields, reduction in volume of the left and right subicula was observed in familial high risk persons with first-degree relatives suffering from SZ or schizoaffective disorder (Francis et al., 2013). The subibulum could mediate hippocampal-cortical interaction, and is purportedly involved in spatial information processing and memory (O’Mara et al., 2009). In the aforementioned study, verbal memory was impaired and significantly correlated with the subicular volume within the relatives of SZ patients (Francis et al., 2013). Dysconnectivity between DLPFC and hippocampal formation has also been reported in SZ patients (Liu et al., 2014). Accordingly, compromised anterior cingulate cortico-hippocampal connection links with the risk of developing SZ in individuals at familial high risk.

Moreover, aberrant DLPFC connectivity and familial risk for SZ are closely related in SZ pathophysiology (Hao et al., 2009; Whitfield-Gabrieli et al., 2009; Woodward et al., 2009; Jang et al., 2011; Rasetti et al., 2011; Su et al., 2013). The prefrontal cortex (PFC) is a compartment of the human brain involved in highly diverse processes, ranging from cognition, motivation, emotion, working memory and complex motor activity to social interactions (Ku et al., 2015; Zhou et al., 2015). These aforementioned results in SZ patients and their relatives suggest that neuro-integrative deficits from the DLPFC to other brain regions are likely to be involved in cognitive function and the familial risk for SZ. However, we did not detect significantly different DLPFC-related connectivity in the sample of relatives of SZ patients in our current study. Last but not least, altered caudate nucleus-related connections were not observed in SZ relatives compared to HCs, either. Unaffected relatives from mixed families (with at least one relative with SZ and one with bipolar disorder) showed reductions in bilateral caudate gray matter density (McIntosh et al., 2004). Paradoxically, our results did not show aberrant connections involving caudate nucleus in relatives of SZ patients. This divergence in findings (i.e., the failure to observe anomalies of connections involved in DLPFC and caudate) could be due to differences in subject selection. In our present study, individuals at high risk for SZ were unaffected first-degree relatives of first episode drug-naïve patients with this illness, rather than mixed first- and second-degree relatives of treated patients commonly used previously. A possible interpretation is the heritable characteristics of SZ and featured effects of facing patients with diverse symptoms before receiving therapy on these subjects in the current study.

We acknowledge that there were several limitations. First, we enrolled a not so large sample size of subjects in this study. Larger sample is desirable to confirm our present findings. Second, the present study did not involve any behavioral data, i.e., we did not measure the severity of cognitive impairment in the relatives. Currently, we are collecting the behavioral data to clarify the relationship between neuroimaging findings and altered cognition. Third, although a recent study demonstrated both noisy and neural effect of head motion on functional connectivity analysis (Zeng et al., 2014), the current study did not examine the difference of head motion between these two groups. This factor should be taken into account in future research.

Our findings show the pattern of effective connectivity among DLPFC, ACC, hippocampus, and caudate in the familial high risk population of SZ patients, which may be tied to a familial risk of SZ. Specifically, we found that increased effective connectivity from the left ACC to right hippocampus and decreased effective connectivity from the right ACC to right hippocampus in unaffected first-degree relatives of first episode SZ patients. The anterior cingulate cortico-hippocampal dysconnectivity may therefore serve as a potential sign of a general vulnerability to develop SZ.

## Author Contributions

HY had full access to all the data in the study and take responsibility for the integrity of the data and the accuracy of the data analysis. Y-BX, CL and L-BC contributed equally to this work. Study concept and design: H-NW, HY. Acquisition, analysis, or interpretation of data: Y-BX, CL, L-BC, JL, FG, LL, T-TL, KL, GC, MX, H-NW, HY. Drafting of the manuscript: Y-BX, CL, L-BC. Critical revision of the manuscript for important intellectual content: Y-BX, CL, L-BC, FG, H-NW, HY. Statistical analysis: CL, L-BC. Administrative, technical, or material support: CL, L-BC, MX. Study supervision: HY.

## Conflict of Interest Statement

The authors declare that the research was conducted in the absence of any commercial or financial relationships that could be construed as a potential conflict of interest.

## Footnotes

^1^http://www.fil.ion.ucl.ac.uk/spm/software/spm8/

^2^http://www.mni.mcgill.ca/

^3^http://www.nitrc.org/projects/wfu_pickatlas/
